# A novel role of FoxO3a in the migration and invasion of trophoblast cells: from metabolic remodeling to transcriptional reprogramming

**DOI:** 10.1186/s10020-022-00522-4

**Published:** 2022-08-08

**Authors:** Hao Chen, Shi-Han Wang, Chang Chen, Xin-Yang Yu, Jia-Nan Zhu, Toby Mansell, Boris Novakovic, Richard Saffery, Philip N. Baker, Ting-Li Han, Hua Zhang

**Affiliations:** 1 Department of Obstetrics and Gynecology, The First Affiliated Hospital of Chongqing Medical University, Chongqing, 400016 China; 2 Canada-China-New Zealand Joint Laboratory of Maternal and Fetal Medicine, Chongqing Medical University, Chongqing, China; 3 The Chongqing Key Laboratory of Translational Medicine in Major Metabolic Diseases, Chongqing, China; 4 Institute of Life Sciences, Chongqing Medical University, Chongqing, China; 5 Department of Obstetrics and Gynecology, The Second Affiliated Hospital of Chongqing Medical University, Chongqing, 400010 China; 6 Molecular Immunity, Murdoch Children’s Research Institute and Department of Paediatrics, University of Melbourne, Melbourne, VIC Australia; 7 College of Medicine, Biological Sciences and Psychology, University of Leicester, Leicester, UK

**Keywords:** Preeclampsia, Forkhead box O3a protein, Aromatic amino acid, Long-chain unsaturated fatty acid, Migration

## Abstract

**Background:**

The forkhead box O3a protein (FoxO3a) has been reported to be involved in the migration and invasion of trophoblast, but its underlying mechanisms unknown. In this study, we aim to explore the transcriptional and metabolic regulations of FoxO3a on the migration and invasion of early placental development.

**Methods:**

Lentiviral vectors were used to knock down the expression of FoxO3a of the HTR8/SVneo cells. Western blot, matrigel invasion assay, wound healing assay, seahorse, gas-chromatography-mass spectrometry (GC–MS) based metabolomics, fluxomics, and RNA-seq transcriptomics were performed.

**Results:**

We found that FoxO3a depletion restrained the migration and invasion of HTR8/SVneo cells. Metabolomics, fluxomics, and seahorse demonstrated that FoxO3a knockdown resulted in a switch from aerobic to anaerobic respiration and increased utilization of aromatic amino acids and long-chain fatty acids from extracellular nutrients. Furthermore, our RNA-seq also demonstrated that the expression of COX-2 and MMP9 decreased after FoxO3a knockdown, and these two genes were closely associated with the migration/invasion progress of trophoblast cells.

**Conclusions:**

Our results suggested novel biological roles of FoxO3a in early placental development. FoxO3a exerts an essential effect on trophoblast migration and invasion owing to the regulations of COX2, MMP9, aromatic amino acids, energy metabolism, and oxidative stress.

## Background

Dysfunctional placentation increases the risk of adverse outcomes of mother and fetus in late gestation (Yang et al. 2017). In the early stage of vascular recasting, trophoblasts invade the spiral artery wall, and vascular endothelial cells are gradually replaced by trophoblast cells (Brosens et al. 2011; Burton et al. 2002; Xu et al. 2018). These allow the vascular cavity to expand, and subsequently, enlarged vascular diameter enables the adequate blood flow and perfusion of the placenta (Yang et al. 2017). Once dysfunction of trophoblast cells appears, it will cause vascular remodeling disorder and superficial invasion, which results in placental dysplasia. Especially, placental disorders may increase the likelihood of preeclampsia (PE), fetal growth restriction (FGR), and recurrent miscarriage in pregnant women (Brosens et al. 2011; Hemberger et al. 2020).

The forkhead box O3a (FoxO3a), which is a member of the forkhead protein factor family (Foxes) (Zaheer et al. 2007), is widely expressed in different tissues and organs, including the heart, placenta, vascular endothelium, and fat (Hedrick et al. 2012; Zhang et al. 2006). FoxO3a involves many cell biological processes, such as cell migration, invasion, metabolism, autophagy, anti-oxidative stress, and apoptosis (Carlsson and Mahlapuu 2002; Warr et al. 2013). There was evidence that FoxO3a promoted the activity of gene networks involved in long-chain fatty acids biosynthesis and catabolism to promote longevity by cooperating with other transcription factors at the gene promoter level (Amrit et al. 2016; Dansen et al. 2004). Furthermore, it has been reported that long-chain fatty acids, especially arachidonic acid (AA), were associated with migration and invasion of cancer (Szymczak et al. 2008). Cyclooxygenase-2 (COX-2) is excessively expressed in many human carcinomas and converts the AA to prostaglandin E2 (PGE2), which promotes metastasis of tumors (Cui et al. 2012). On the other hand, increasing evidence illustrated the inseparable negative correlation between aromatic amino acids (tyrosine, phenylalanine, tryptophan) and cancer metastasis (Hasim et al. 2013, 2012). Aromatic amino acids are related to COX-2-mediated migration and invasion (Cheng et al. 2012; Gu et al. 2021). Although many studies pinpoint that FoxO3a plays an essential regulatory role in long-chain fatty acids and amino acids metabolism, the potential mechanism of how FoxO3a modulates these metabolites to mediate trophoblast migration and invasion remains unknown.

Hence, the purpose of this study is to investigate the role of FoxO3a in the HTR8/SVneo cell line and the related mechanism on long-chain fatty acids and amino acids metabolism that are associated with migration and invasion, hoping to understand the regulatory mechanism of FoxO3a in early placental development.

## Methods and materials

### Cell culture and cell knockdown

The cells involved in the experiment were immortalized human trophoblast HTR8/SVneo cell line obtained from the American Type Culture Collection (ATCC, Manassas, VA, USA). This is the recommended cell model to explore both the migration and invasion capability in early placental development (Novakovic et al. 2011). The cells were cultured in the RPMI 1640 medium containing 10% fetal bovine serum (FBS, PAN, Germany) and 1% penicillin and streptomycin and incubated in a standard cultivation environment (in a humidified environment with 37 °C and 5% CO_2_). Lentiviral vectors (GenePharma, China) were used to transfect HTR8/SVneo cells for 48 h to knock down the expression of FoxO3a, and there were two cell groups: a negative control group (sh-NC) and a scrambled shRNA group (sh-FoxO3a). Sodium nitroprusside (SNP) was selectively added into the complete medium to construct oxidative stress in the cells, and the optimal concentration has been explored in previous studies.

### Western blot

Cellular protein from the transfected HTR8/SVneo cells was extracted using a RIPA lysis buffer (Beyotime Biotechnology, China) containing PMSF (1:100, Beyotime Biotechnology, China). The concentration of the extracted protein was determined by using a BCA assay kit (Beyotime Biotechnology, China). Each protein sample was loaded onto SDS-PAGE for electrophoresis and then transferred to a piece of PVDF membrane. TBST containing 5% skimmed milk was used to block the PVDF membrane for 1 h. Next, the membrane was incubated with various primary rabbit antibodies, including anti-FoxO3a (1:800, Catalog#: 12829, Cell Signaling Technology) and β-actin (1:5000, Catalog#: GB11001, Servicebio) at 4 °C overnight. After incubating with goat anti-rabbit IgG for 1 h, bands density was detected using the Quantity One System image analyzer (Bio-Rad, USA).

### Cell migration assay

Wound Healing Assay was performed to assess the ability of migration between the sh-NC group and the sh-FoxO3a group. 5 × 10^5^ cells were plated into a 6-well plate. A 200 µl sterile pipette tip was applied to scratch the cell monolayers when cells reached 90% confluence. Floating cells were removed with PBS, then the cells were incubated in fresh complete mediums for 24 h. Images were taken by microscopy (EVOS FL Auto Imaging System, Life Technologies, USA) at 0 h and 24 h after the scratch, and ImageJ software was used to measure the wound healing rate.

### Cell invasion assay

The invasiveness of the two group cells was detected using Matrigel invasion assay. After the diluted matrigel (BD BioScience) was added to the invasion chamber for 4 h, about 5 × 10^4^ cells were seeded into the upper compartment. After incubation for 24 h, a cotton swab was used to wipe the residual cells in the upper chamber. The lower chamber cells were fixed using 4% paraformaldehyde, washed with PBS, and stained with crystal violet (Beyotime Biotechnology, China). We used microscopy (EVOS FL Auto Imaging System, Life Technologies, USA) to determine the amount of the cells on the lower chamber. ImageJ software was used to evaluate the invasion rate.

### Metabolic flux analysis of the mitochondrial

Seahorse XFp Analyzer (Agilent, Santa Clara, CA) was used to evaluate the oxygen consumption rate (OCR), which reflected the mitochondrial function. HTR8/SVneo cells (Sh-NC group, sh-FoxO3a group, Sh-NC + SNP group, sh-FoxO3a + SNP group) were seeded in Seahorse XFp plates and cultured in a complete medium overnight. The next day, XF assay medium was added to replace the complete medium, and then the cells were incubated at 37 °C without CO_2_ input atmosphere. Oligomycin (working concentration: 2.5 μM), FCCP (working concentration:1.0 μM), and Rotenone (working concentration: 0.5 μM) were added into the probe separately. At the time point of 26 min (basal respiration detection lasted for 26 min), 50 min and 70 min, oligomycin (25 μM), FCCP (10 μM), antimycin A (5 μM) /rotenone (5 μM) were injected into the chamber respectively. Mitochondrial parameters (basal respiration, proton spill, maximal respiration, and ATP turnover rate) were evaluated by various OCR indexes. Seahorse XFp software was used to analyze the OCR index.

### Intracellular, extracellular, and biomass metabolite extraction from cell culture

2 ml of each culture medium of sh-NC and knockdown group HTR8/SVneo cells was used for extracellular chemical derivatization. For the intracellular metabolite extraction, 10 ml of liquid nitrogen was added to each plate of HTR8/SVneo cells. Then cold methanol/chloroform (9:1), containing the standard internal 2,3,3,3-d4-alanine (0.3 µmol), was used to extract metabolite from HTR8/SVneo cells. The collected samples were centrifuged at 15,000 g for 15 min at 4 °C, and the supernatant and the biomass were obtained. The supernatant attained was dried in the SpeedVac (Labconco Corp., Missouri, USA) for 5 h at room temperature and stored at − 80 °C for intracellular chemical derivatization. For the biomass metabolite extraction, biomass fraction was dissolved in 100 μl sodium hydroxide, and then samples were kept at 98 °C for 10 min. 100 μl ddH_2_O and 200 μl methanol were added to each heated sample. The collected specimens were centrifuged at 15,000 g for 15 min at 4℃, and the supernatant was obtained for chemical derivatization.

### Chemical derivatization of metabolites and GC–MS assay

The samples from Intracellular, extracellular, and biomass were derivatized using the methyl chloroformate (MCF) method as previously described (Smart et al. 2010). The chemical derivatives were analyzed by a system of Agilent GC7890B coupled to an MSD5977A mass selective detector (EI) set at 70 eV. The ZB-1701 GC capillary column (30 m × 250 µm id × 0.15 µm with 5 m guard column, Phenomenex) was used for metabolite analysis. The parameter analysis was previously described (Smart et al. 2010).

### GC–MS data analysis

The software, which is based on MassOmics XCMS R, was applied to extrapolate the relative abundance of the metabolites through the peak height of the most enriched ion mass (https://zenodo.org/record/4961895). To achieve stable repeatability and instrumental deviations and minimize sample preparation, the corresponding concentration of the identified metabolites were normalized by an internal standard (D4-alanine), total ion concentration of the cellular metabolome, and revised by quality control of pooled samples. Before the HTR8/SVneo metabolome was analyzed, each metabolite concentration was transformed by log_10_ scale and Pareto scaling set up Gaussian distribution for this data. Model validation and partial least squares discriminant analysis (PLS-DA) were operated through MetaboAnalyst 5.0 (https://www.metaboanalyst.ca/). The Student's *t*-test and a false discovery rate were implemented to calculate the significance of HTR8/SVneo metabolites between two groups by using R software. Only two-tailed *P*-values less than 0.05 were regarded as statistically significant. Receiver operating characteristic (ROC) curves were conducted using the pROC R package (Robin et al. 2011). Pathway enrichment analysis was performed by blasting our identified metabolites to the Kyoto Encyclopedia of Genes and Genomes (KEGG) database. The chord plot illustrated how the GOplot R package rendered metabolites participating in KEGG metabolic pathways.

### Isotope tracer experiment

The effect of 13C-labeled tracer (U-^13^C_6_ glucose) on flux estimation precision was measured in previous research (Han et al. 2019). There were two types of culture mediums used in ^13^C-glucose isotope labeling and metabolomics experiments: (1) RPMI 1640 medium containing 30% ^13^C_6_-labelled glucose (U-^13^C_6_ glucose); (2) RPMI 1640 medium containing 30% ^12^C_6_-labelled glucose. Subsequently, as described above, metabolite extraction, chemical derivatization, and GC–MS analysis were performed.

### Cellular oxidative stress detection

ROS in the HTR8/SVneo cells was measured using a Fluorometric Intracellular ROS Kit (MAK143, Sigma-Aldrich). After knockdown of FoxO3a by lentiviral vectors (GenePharma, China) for 2 days, the HTR8/SVneo cells seeded in six-well plates were incubated with 100 μl of master reaction mix at 37 °C for 30 min. Subsequently, green fluorescence was evaluated using a fluorescence microscope or measured at λex490 nm/λem520 nm with a microplate reader.

### RNA sequencing

According to the manufacturer's protocol, total RNA was extracted from HTR8/SVneo cells when it reached 90% fusion with TRIzol (Invitrogen, Carlsbad, CA, USA). Then, Agilent 2100 Bioanalyzer (Agilent Technologies, Palo Alto, CA, USA) was used to assess the quantity of RNA and then qualified RNA was detected by performing agarose gel electrophoresis (RNase free). Next, beads of Oligo(dT) were applied to enrich mRNA. The enriched RNA fragments were broken by ultrasound and then as templates to synthesize cDNA with random primers. Before the purified cDNA came to Illumina, sequencing adapters, end-repaired, and poly(A) added were performed using a PCR extraction kit (Qiagen, Venlo, The Netherlands). RNase-free agarose gel electrophoresis was operated for size selection of the ligation products. Next, fragments amplified by PCR were sequenced using Illumina HiSeq2500 by Gene Denovo Biotechnology company (Guangzhou, China). Differential expression of the obtained RNA (two groups) was calculated by DESeq2 and edgeR software. Only the false discovery rate (FDR) below 0.05 and fold change ≥ 2 of these transcripts were considered differentially expressed genes.

### qRT-PCR

According to the manufacturer's instructions, RNA of the two groups of cultured cell lines was extracted using TRIzol reagent (Invitrogen, USA). The obtained RNA concentration was assessed using ultraviolet spectroscopy (Nano Drop 2000, Thermo, USA). Subsequently, Roche Reverse Transcription Kit (#07912455001, Roche, Germany) transcribes 1 μg RNA of each sample to cDNA reversely. GAPDH (housekeeping gene) was used to control relative gene expression analysis. The primer pairs of GAPDH were: forward: 5′ GGAAGCTTGTCATCAATGGAAATC 3′, reverse: 5′ TGATGACCCTTTTGGCTCCC 3′. Primers for the target gene were as follows: COX-2: forward: 5′ AAGACAGATCATAAGCGAGGGC 3′, reverse: 5′ AAACCGTAGATGCTCAGGGACT 3′; MMP9: forward: 5′ TCGACGTGAAGGCGCAGAT 3′, reverse: 5′ AGAAGCGGTCCTGGCAGAAATA 3′.

## Results

### Depletion of the expression of FoxO3a in HTR8/SVneo cells.

**Fig. 1:**
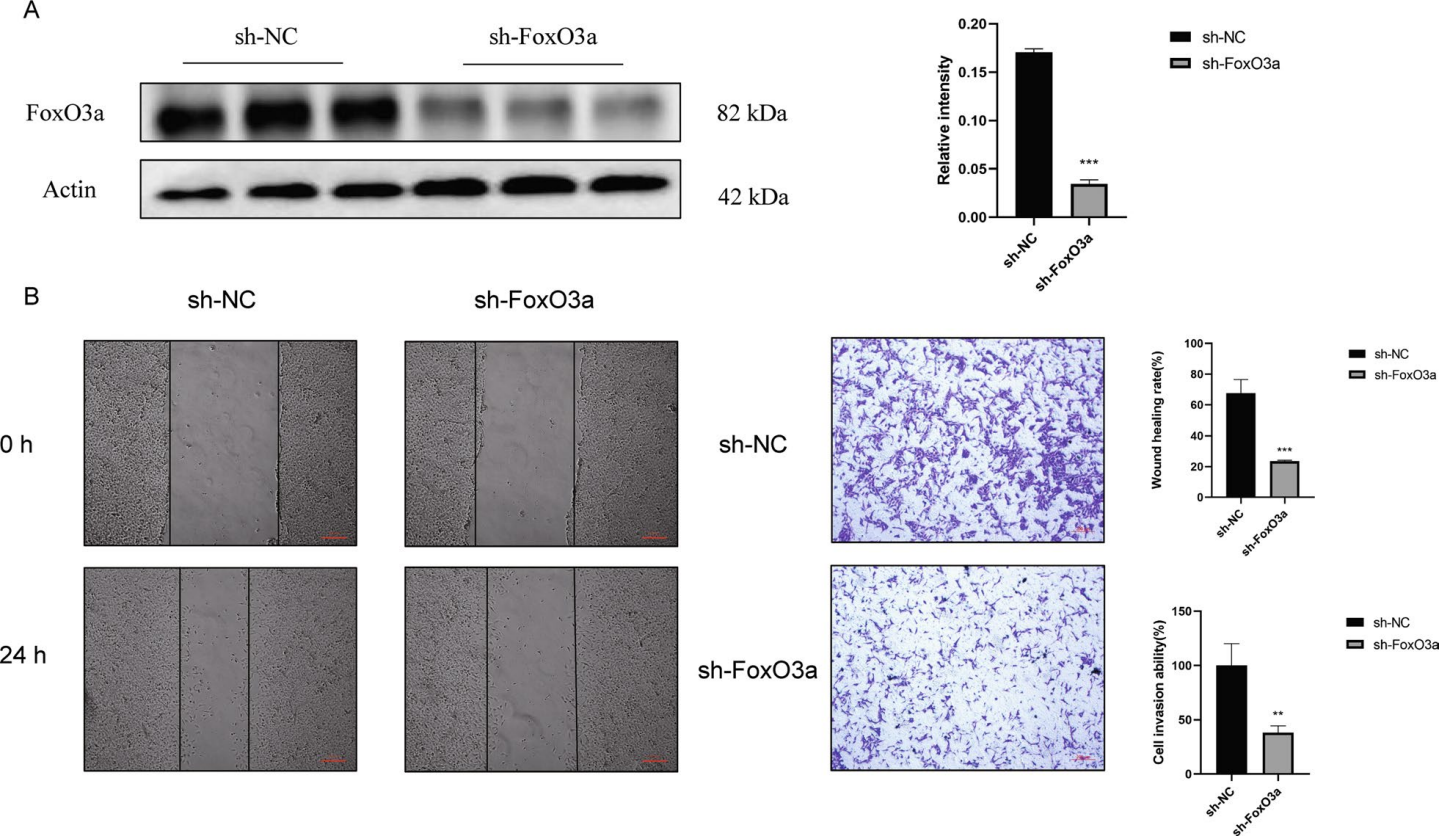
Detection of transfection efficiency of lentivirus targeting FoxO3a in HTR8/SVneo cells and the abilities of migration and invasion of HTR8/SVneo cells after FoxO3a depletion. **A** After 48 h transfection of lentivirus, the expression of FoxO3a in HTR8/SVneo cells was analyzed by Western Blot. **B** Wound-healing assay and Matrigel transwell assay illustrated migration and invasion abilities after FoxO3a depletion. Results are shown as mean ± SEM, n = 3, **P < 0.01 and ***P < 0.001

The expression of FoxO3a was depleted by lentiviral transfection to explore how FoxO3a regulates the biological process and prepare for subsequent experiments. Our results demonstrated that the expression of FoxO3a was distinctly down-regulated in the knockdown group with a transduction efficiency of over 90% (Fig. 1A). Thus, FoxO3a has been successfully knocked down.

### FoxO3a deletion inhibited the migration/invasion process of HTR8/SVneo cells

We performed Wound Healing Assay and Matrigel cell invasion assay to investigate the effect of sh-FoxO3a on HTR8/SVneo cells. It was shown that the wound healing rate (the ability of migration, Fig. 1B) and Matrigel cell invasion (the ability of invasion, Fig. 1B) of the sh-FoxO3a group decreased compared with the sh-NC group. Therefore, FoxO3a knockdown led to poor migration and invasion.

### Differences in intracellular metabolite and biomass profiles between the sh-NC and sh-FoxO3a groups

**Fig. 2:**
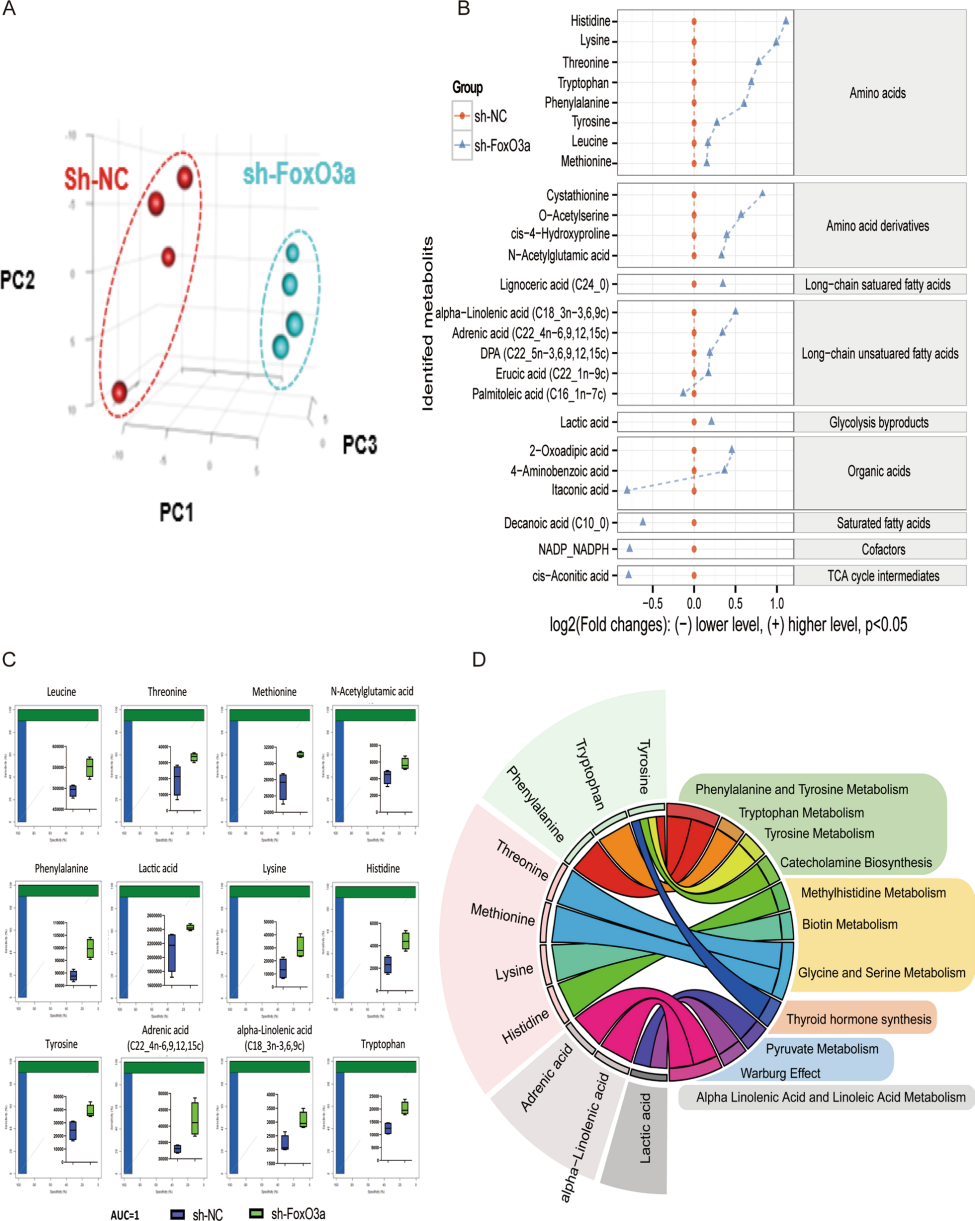
The characteristics of the intracellular metabolic data. **A** partial least square discriminant analysis (PLS-DA) between the sh-NC group and sh-FoxO3a group. **B** The student t-test analysis showed that 25 metabolites differed between the sh-NC and knockdown groups. **C** The area under the receiver operating characteristic (ROC) curve for intracellular metabolites. There were 12 metabolites under the ROC curve greater than 90%, mainly amino acids and long-chain unsaturated fatty acids. **D** The R package of PAPi. Only statistically significant differences in metabolites and low false discovery rate (*P* < 0.05 by the Student's *t*-test) are illustrated

**Fig. 3:**
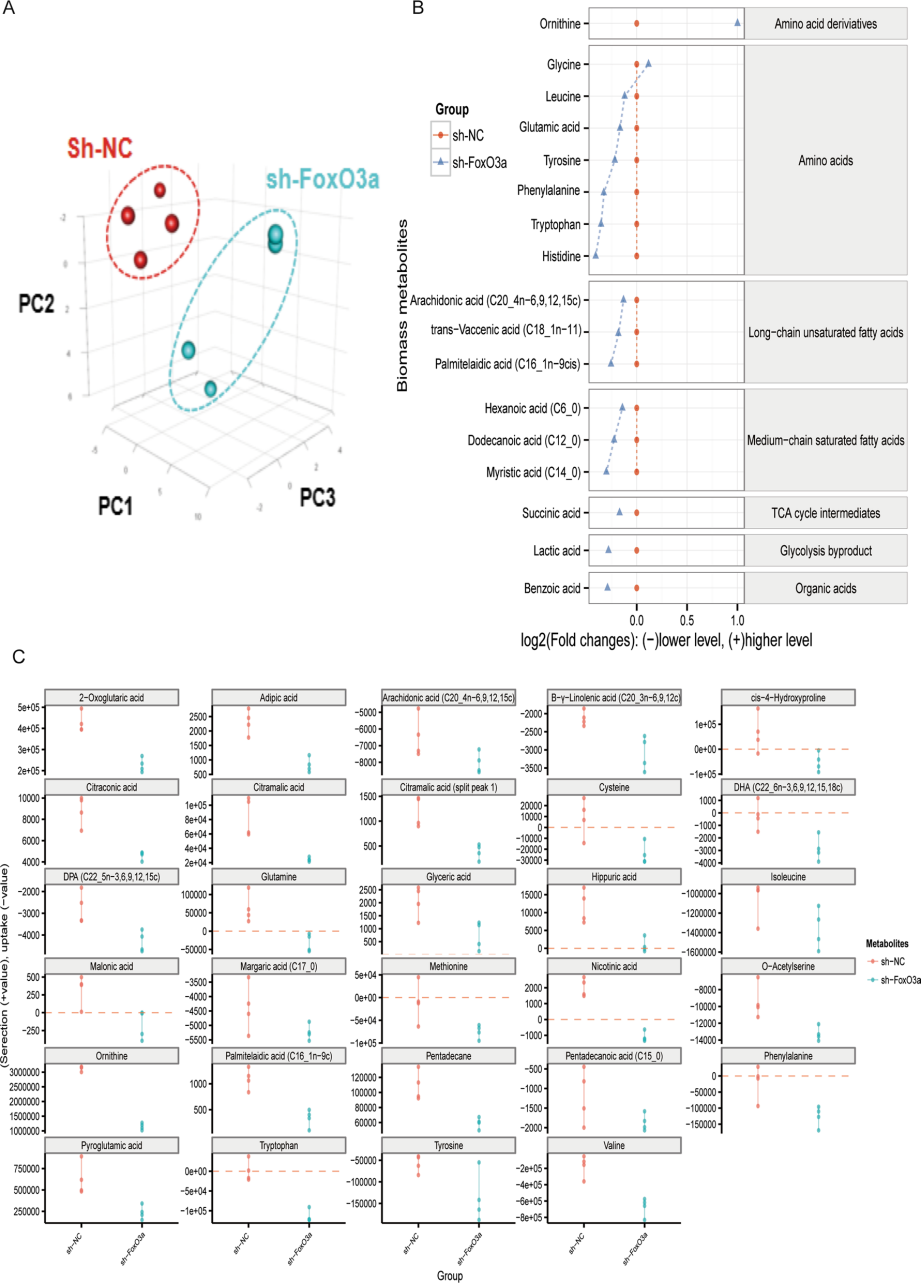
The characteristics of the biomass and the extracellular metabolic data. **A** PLS-DA between the sh-NC group and sh-FoxO3a group. **B** The Student's *t*-test analysis showed that 20 metabolites differed between the sh-NC and the knockdown groups. **C** Extracellular metabolites difference between the sh-NC group and sh-FoxO3a group. Above the red line (positive values) indicates secretion; below (negative values) means absorption. Only statistically significant differences in metabolites and low false discovery rate (*P* < 0.05 by the Student’s *t*-test) are illustrated

To explore how the FoxO3a gene influences metabolic changes, we compared the difference in intracellular metabolites and biomass between the control and knockdown groups. For intracellular metabolites, there were 347 GC–MS peaks detected in the sh-NC group and sh-FoxO3a group, and 242 of which were identified by our MCF mass library. We used partial least square discriminant analysis (PLS-DA) to diversify the characteristics of the metabolic data of GC–MS, and the result revealed that the sh-NC group and the sh-FoxO3a group were clustered separately (Fig. 2A). The univariate analysis showed that 25 metabolites differed distinctly between the sh-NC and sh-FoxO3a groups (*P* < 0.05; Fig. 2B). The abundance of most metabolites increased in the sh-FoxO3a group, and only a few metabolites, including NADP_NADPH, Palmitoleic acid (C16_1n-7c), Itaconic acid, Decanoic acid (C10_0), and cis-Aconitic acid, decreased (Fig. 2B). Changes in these metabolite ratios in two groups may likely result from the migration and invasion. Interestingly, 12 metabolites under the receiver operating characteristic (ROC) curve were greater than 90%, including eight amino acids, one amino acid derivative, two long-chain unsaturated fatty acids, and one glycolysis byproduct (Fig. 2C). There were 89 GC–MS peaks detected in the two groups for biomass profiles, which our MCF mass library identified. PLS-DA was used to diversify the characteristics of the biomass data, and the result revealed that the sh-NC group and the sh-FoxO3a group were clustered separately (Fig. 3A). The univariate analysis illustrated that 17 metabolites differed distinctly between the sh-NC and sh-FoxO3a groups (*P* < 0.05; Fig. 3B). Almost all amino acids and long-chain fatty acids were down-regulated in the FoxO3a knockdown group. Thus, the depletion of FoxO3a resulted in amino acids and long-chain fatty acids accumulation inside the cells.

### The differential concentration of extracellular metabolic profiles between the sh-NC and sh-FoxO3a groups

We further detected the metabolite differences in the medium to support our intracellular metabolic profile. The results were contrary to the trend of intracellular metabolite differences. We found that FoxO3a knockout reduced extracellular secretion or promoted extracellular metabolites absorption, which is inconsistent with the intracellular findings. The above red line (positive values) indicates secretion, which means absorption is below the red line (negative values). As we can see, tyrosine, phenylalanine, and tryptophan owned a lower concentration in the medium after FoxO3a depletion (Fig. 3C). Thereby, the reduction of extracellular aromatic amino acids is likely uptaken by cells with FoxO3a knockdown.

### Effect of FoxO3a gene knockdown on the intracellular metabolic state of HTR8/SVneo cells

The R package of Pathway Activity Profiling (PAPi) was used to generate a metabolic activity profile based on the intracellular metabolites of the control and knockdown groups. It was shown that 11 metabolic pathways were significantly enriched, including phenylalanine and tyrosine metabolism, tryptophan metabolism, tyrosine metabolism, catecholamine biosynthesis, methylhistidine metabolism, biotin metabolism, thyroid hormone synthesis, pyruvate metabolism, Warburg effect, and alphalinolenic acid/linoleic acid metabolism (*P* < 0.05; Fig. 2D). Hence, this data indicated that FoxO3a knockdown mainly influences the metabolism of aromatic amino acids, long-chain fatty acids, and glycolysis.

### The metabolite distribution profile of 30% ^13^C6-labelled glucose or 30% ^12^C6-labelled glucose is the only carbon source for HTR8/SVneo cells

**Fig. 4.**
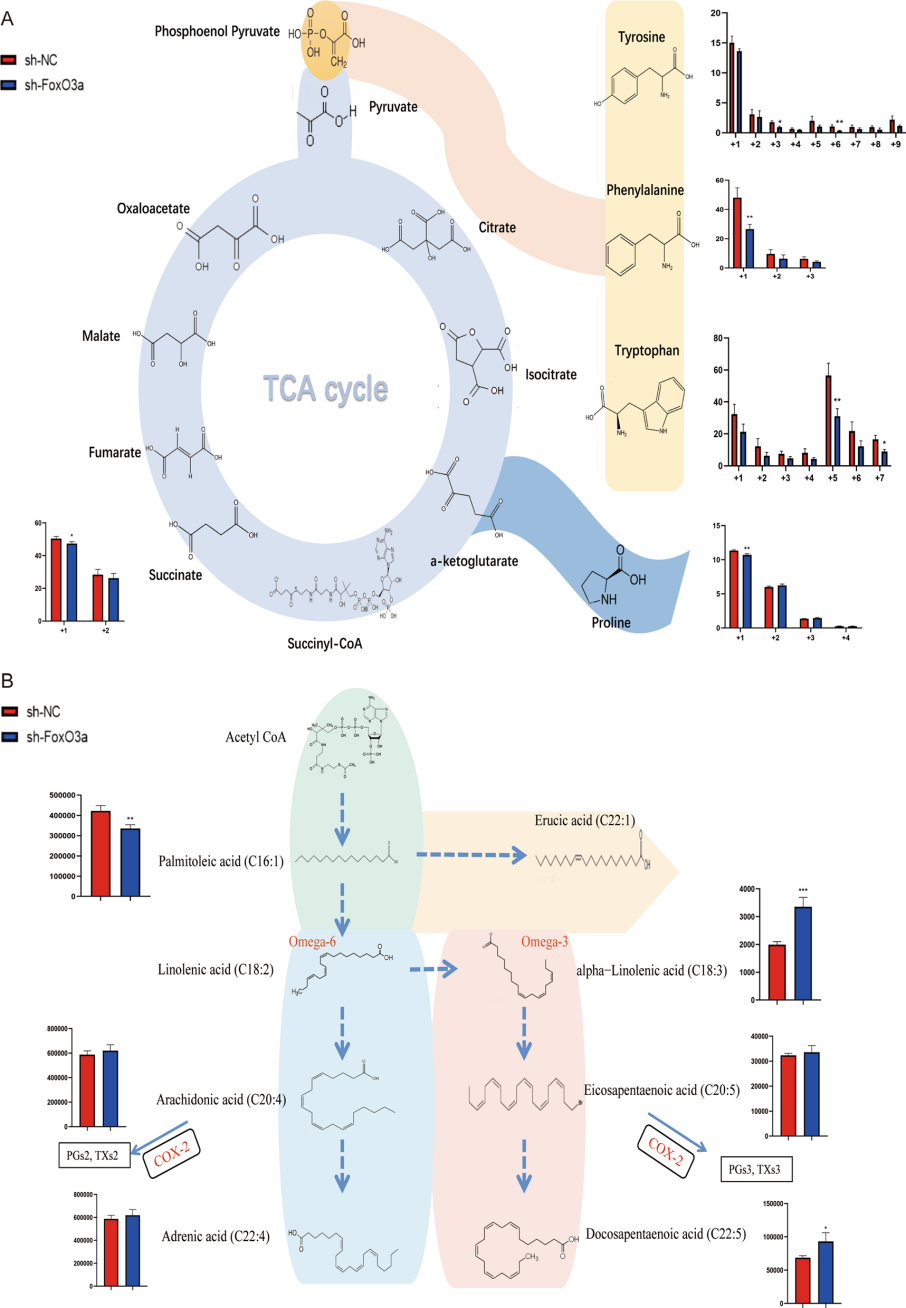
^13^C-labelled glucose metabolic flux and intracellular long-chain unsaturated fatty acids metabolism differences and Bioenergetics by Seahorse XFp metabolic flux analysis. **A** Tyrosine, phenylalanine, and tryptophan owned a lower rate of biochemical conversion after FoxO3a depletion. **B** Our intracellular metabolic result showed differences in long-chain unsaturated fatty acids between sh-NC and knockdown groups, and differential metabolites mostly belong to omega-6 or omega-3 fatty acids. Omega-3 and omega-6 fatty acids are mainly involved in inflammation and migration progress. Results are shown as mean ± SEM, n = 3, *P < 0.05, **P < 0.01 and ***P < 0.001

To track how the knockdown of the gene FoxO3a affects the cell metabolism profile, we used ^13^C-labelled glucose as the only carbon source to provide cells in the control group and knockdown group. Our principle was that the labeled carbon atoms of a metabolite increase, proving that the labeled carbon source is converted to it. In short, the labeling metabolite owns a higher rate of biochemical conversion. As we can see, tyrosine, phenylalanine, and tryptophan owned a lower rate of biochemical conversion after FoxO3a depletion, while proline and succinate were contrary (Fig. 4A). Consistently, flux analysis also supported that intracellular aromatic amino acids are absorbed from the medium in the sh-FoxO3a group.

### sh-FoxO3a restrained the respiration of HTR8/SVneo cells and elevated intracellular ROS

**Fig. 5:**
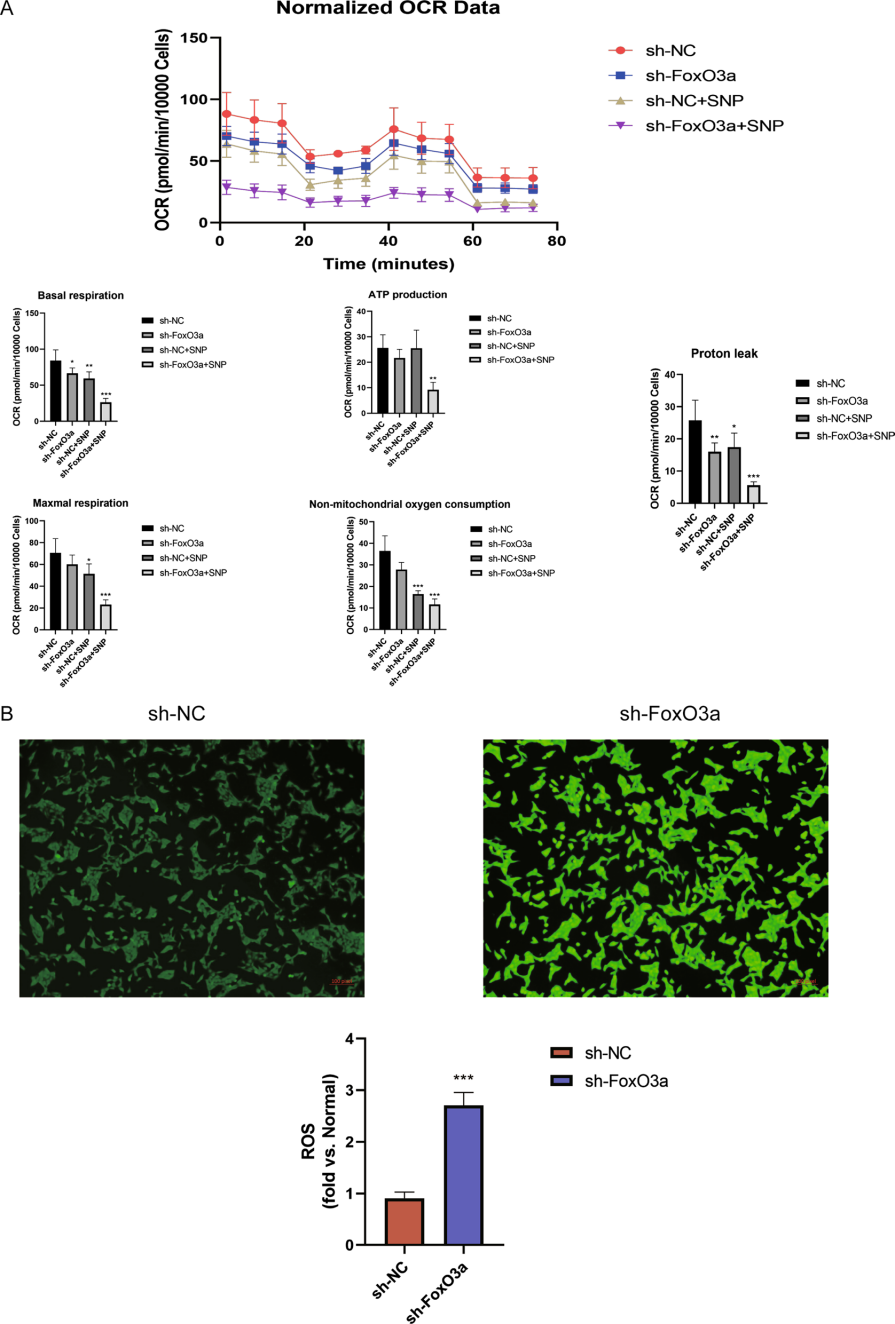
Bioenergetics by Seahorse XFp metabolic flux analysis and the oxidative stress analysis. **A** FoxO3a depletion group exhibited a distinct reduction in basal respiration and proton leak. **B** FoxO3a knockdown-induced oxidative stress. Intracellular ROS was measured using a fluorescence microscope (Scale bar = 400 um) and fluorescence intensity was measured at λex490 nm/λem 520 nm with a microplate reader. Results are shown as mean ± SEM, n = 3, *P < 0.05, **P < 0.01 and ***P < 0.001

To investigate whether FoxO3a was involved in regulating mitochondrial respiration, we performed a seahorse assay to test the mitochondrial oxygen consumption rate (OCR). Compared to the sh-NC group, the sh-FoxO3a group, sh-NC + SNP group, and sh-FoxO3a + SNP group exhibited a distinct reduction in basal respiration and proton leak, which was reflectedin decreased oxygen consumption (Fig. 5A). For ATP production, only the group of sh-FoxO3a + SNP showed a significant loss versus the control group (Fig. 5A). Under the condition of oxidative stress caused by SNP, both the control group and the knockdown group displayed a definite reduction in maximal respiration and non-mitochondrial oxygen consumption (Fig. 5A). Therefore, we assumed that there was increased oxidative stress in the cells. We performed a ROS activity assay using a Fluorometric Intracellular ROS Kit(MAK143, Sigma-Aldrich) to investigate whether FoxO3a knockdown induced oxidative stress. The results showed that ROS accumulated in the cells after FoxO3a depletion (Fig. 5B). Thus, FoxO3a knockdown restrained HTR8/SVneo cells' respiration while elevated intracellular ROS.

### The migration was the most differential biological process after sh-FoxO3a knockdown in HTR8/SVneo cells

**Fig. 6:**
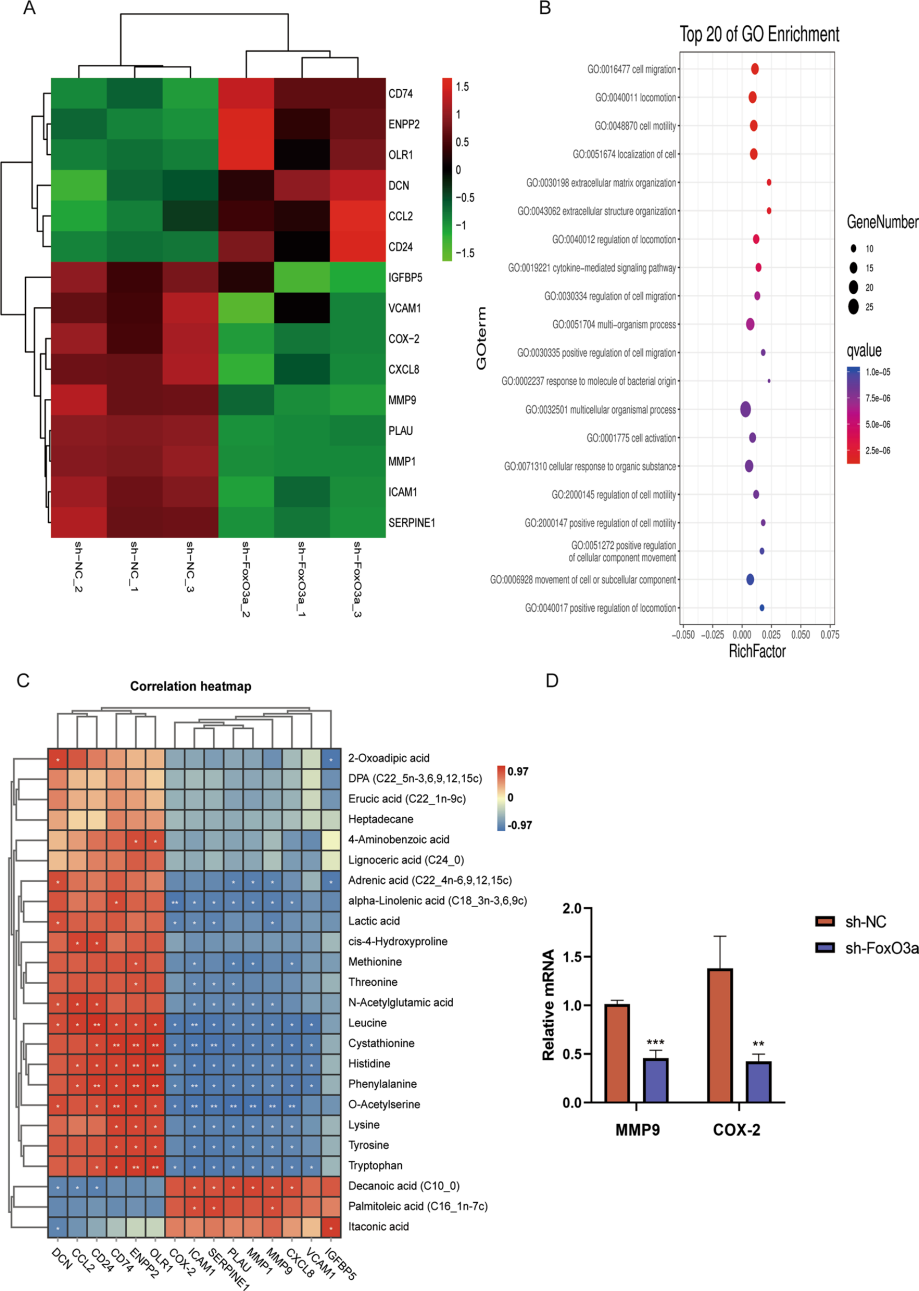
FoxO3a regulates HTR8/SVneo cells migration progress. **A** The heatmap illustrates the copy numbers of mRNAs. Red represents higher transcription, and green represents lower transcription. **B** Enriched Gene Ontology (GO) analysis illustrated that migration of HTR8/SVneo cells was the most affected biological process after FoxO3a exhaustion. **C** Correlation analysis between transcriptomics and metabolomics. Red represents positive correlation, and blue represents negative correlation. **D** qPCR shows reduced expression of the genes (COX-2 and MMP9). Only the results showing statistically significant Student’s t-test (*P* < 0.05) and minimum false discovery rate (q < 0.05) are shown, n = 3, **P < 0.01 and ***P < 0.001

To explore the potential molecular mechanism related to FoxO3a, we performed an RNA-Seq experiment. We found that many genes were upregulated or downregulated (Fig. 6A). When we conducted GO enrichment analysis on these genes, we found that the most influential biological process was migration (Fig. 6C). Subsequently, we performed a correlation analysis between transcriptomics and metabolomics profiles, and we found nine genes positively correlated with significantly differential metabolites (Fig. 6B). qRT-PCR unveiled that the expression of genes COX-2 (also called prostaglandin-endoperoxide synthase 2 (PTGS2)) and MMP9 had decreased (Fig. 6D). Collective evidence demonstrated that FoxO3a knockdown depressed the migration and invasion process of HTR8/SVneo cells by inhibiting the expression of COX-2 and MMP9.

## Discussion

Our research was the first to apply transcriptomic, metabolomic, and isotope labeling experiments to explore the role of FoxO3a in regulating migration and invasion of trophoblast, which is essential for the early development of the placenta. Our data demonstrated that FoxO3a depletion restrained the migration and invasion of HTR8/SVneo cells. These phenotypical phenomena were also accompanied by the metabolic reprogramming of global metabolism and the utilization of extracellular nutrients such as aromatic amino acids and long-chain fatty acids. The migration/invasion process seems to be closely associated with metabolic remodeling and transcriptional reprogramming.

FoxO3a is a major contributing factor for regulating the energy metabolism of the HTR8/SVneo cells. Although our RNA-seq result did not show the expression of key enzymatic genes involved in the switch from oxidative phosphorylation to glycolysis, metabolic flux (Fig. 4A), seahorse (Fig. 5A), and ROS analysis (Fig. 5B) results showed that mitochondrial respiration was diminished along with reduced ATP production and excessive ROS. What is more, the concentration of lactic acid was elevated in response to the FoxO3a knockdown (Fig. 2B). These findings implied that the knockdown of FoxO3a contributed to the impaired TCA cycle and upregulatedglycolysis. Alessia Peserico et al. consistently reported that FoxO3a operated as a protection mechanism to maintain cellular respiration upon metabolic stress and nutrient shortage. Their research illustrated that a transcriptional complex (FoxO3a, SIRT3, and RNA polymerase at DNA regulatory regions of mitochondria) would be activated to protect mitochondrial respiration against oxidative stress (Peserico et al. 2013). Ferber et al. proved that activation of FoxO3a restrained the expression of proteins of mitochondria and levels of respiratory complexes through inhibition of c-Myc, and these prevented ROS production (Ferber et al. 2012). It has also been reported that FoxO3a promoted the expression of the mitochondrial respiratory-related genome to facilitate aerobic respiration for ATP production (Celestini et al. 2018). On the other hand, our transcriptomic data revealed that FoxO3a knockdown reduced the expression of hexokinase domain containing 1 (HKDC1), which is known to suppress gluconeogenesis (Irwin and Tan 2008). Khan et al*.* also demonstrated that hepatic HKDC1 overexpression upon pregnancy reduces gluconeogenesis in mice (Khan et al. 2019; Zapater et al. 2021). Gluconeogenesis seems to be upregulated to fulfill the energy demand by utilizing alternative subtracts to compensate for the inhibition of mitochondrial respiration by FoxO3a. Thus, it could be a potential reason that aromatic amino acids and long-chain fatty acids were uptaken from their external environment.

FoxO3a-mediated migration/invasion of trophoblast seems to be associated with aromatic amino acid and long-chain fatty acid metabolism in early pregnancy. We found that the migration and invasion of HTR8/SVneo cells were compromised after FoxO3a knockdown (Fig. 1). Our intracellular metabolites (Fig. 2), extracellular metabolites (Fig. 3C), biomass (Fig. 3B), and ^13^C-labelled glucose metabolic flux (Fig. 4A) results showed that there were many significantly differential enrichments of amino acids between wildtype and FoxO3a knockdown, particularly aromatic amino acids were accumulated in level and reduced ^13^C flux enrichment intracellularly. Recently, several researchers reported that tryptophan (one of the three aromatic amino acids) had been involved in migration and invasion via cyclooxygenase-2 (COX-2, also named PTGS2) and matrix metallopeptidase 9 (MMP9) (Gu et al. 2021; Liu et al. 2020). Our previous RNA-seq study consistently demonstrated that COX-2 and MMP9 were decreased after FoxO3a depletion (Chen et al. 2021), and the expression of these two genes is negatively correlated with all three aromatic amino acids acid concentrations under FoxO3a knockdown condition (Fig. 6B). Moreover, it has been proposed that melatonin and 5-methoxytryptophan (5-MTP) are downstream metabolites from tryptophan that abrogated P52 binding to κB enhancer elements at COX-2 promoters, thereby inhibiting the COX-2 expression (Cheng et al. 2012; Deng et al. 2006; Wu et al. 2014). Furthermore, COX-2 is a rate-limiting enzyme that mediates the production of prostaglandin E2 (PGE2) from arachidonic acid (AA). Subsequently, PGE2 activates a JAK2/STAT3 pathway to elevate the expression of metallopeptidase 9 (MMP9) (Kawahara et al. 2015; Lee et al. 2020). On the other hand, our intracellular profiles (Fig. 2) showed that many long-chain fatty acids (e.g., arachidonic acid and a-Linolenic acid) were accumulated inside the HTR8/SVneo cells. Although there was no significant difference for arachidonic acid (AA) between the wildtype and the knockdown group in intracellular metabolite profile, the ratio of AA to linolenic acid (a metabolite upstream of AA) and the abundance of adrenic acid (a metabolite downstream of AA) increased compared to the sh-NC group. Recently, a-Linolenic acid (ALA) was reported to suppress migration and invasion in many malignant tumors, such as prostate (du Toit et al. 1996), colon (Chamberland and Moon 2015), and breast (Wiggins et al. 2015). Another study showed that ALA could inhibit cell migration and invasion via decreased expression of COX-2 that mediated the transformation of AA to prostaglandin E2 (PGE2) and then promoted MMP9-mediated migration and invasion (Deshpande et al. 2016). Our RNA-seq analysis illustrated that the expression of COX-2 and MMP9 were inhibited after FoxO3a depletion (Fig. 6A), which may lead to compromised migration and invasion progress via impairing AA metabolism. Therefore, through transcriptomics and metabolomics, we suggested that FoxO3a depletion leads to the accumulation of intracellular aromatic acids and the long-chain fatty acid, resulting in the inhibition of COX-2 and MMP9 mediated migration and invasion of trophoblast.

Aromatic amino acids may promote catecholamine anabolism and eventually contribute to placental oxidative stress. Our metabolic pathway analysis pinpointed that intracellular metabolic flux redirected aromatic amino acids toward catecholamine metabolism (Fig. 2D). Meanwhile, there was excessive ROS accumulated in the cells (Fig. 5B). It is widely accepted that catecholamine is biosynthesized from phenylalanine and tyrosine (Nazari et al. 2020; Végh et al. 2016). There is also evidence that the placenta could synthesize catecholamine from aromatic amino acids, and the abundance of catecholamine in the preeclampsia was significantly increased compared to the normal pregnancy (Turner et al. 2008).

Moreover, the accumulation of catecholamine has been reported to elevate oxygen demand/supply imbalance, blood flow reduction, direct toxic effect, free radical formation, and increased excitotoxicity (Ma et al. 2004), which are the common pathophysiology observed in the dysregulated placenta. In addition, Kajihara et al. demonstrated that FoxO3a also plays an important role in regulating the antioxidant stress process (Kajihara et al. 2006), which should be investigated in a future study. Thus, with the elevation of phenylalanine, tyrosine, and downstream metabolites, there was less blood, oxygen supply and excessive ROS production in the placenta, which may partly contribute to the trophoblast's poor migration/invasion. Thus, the underlying mechanisms of FoxO3a induced aromatic amino acid-catecholamine metabolism should be investigated in early placental development.

## Conclusions

**Fig. 7:**
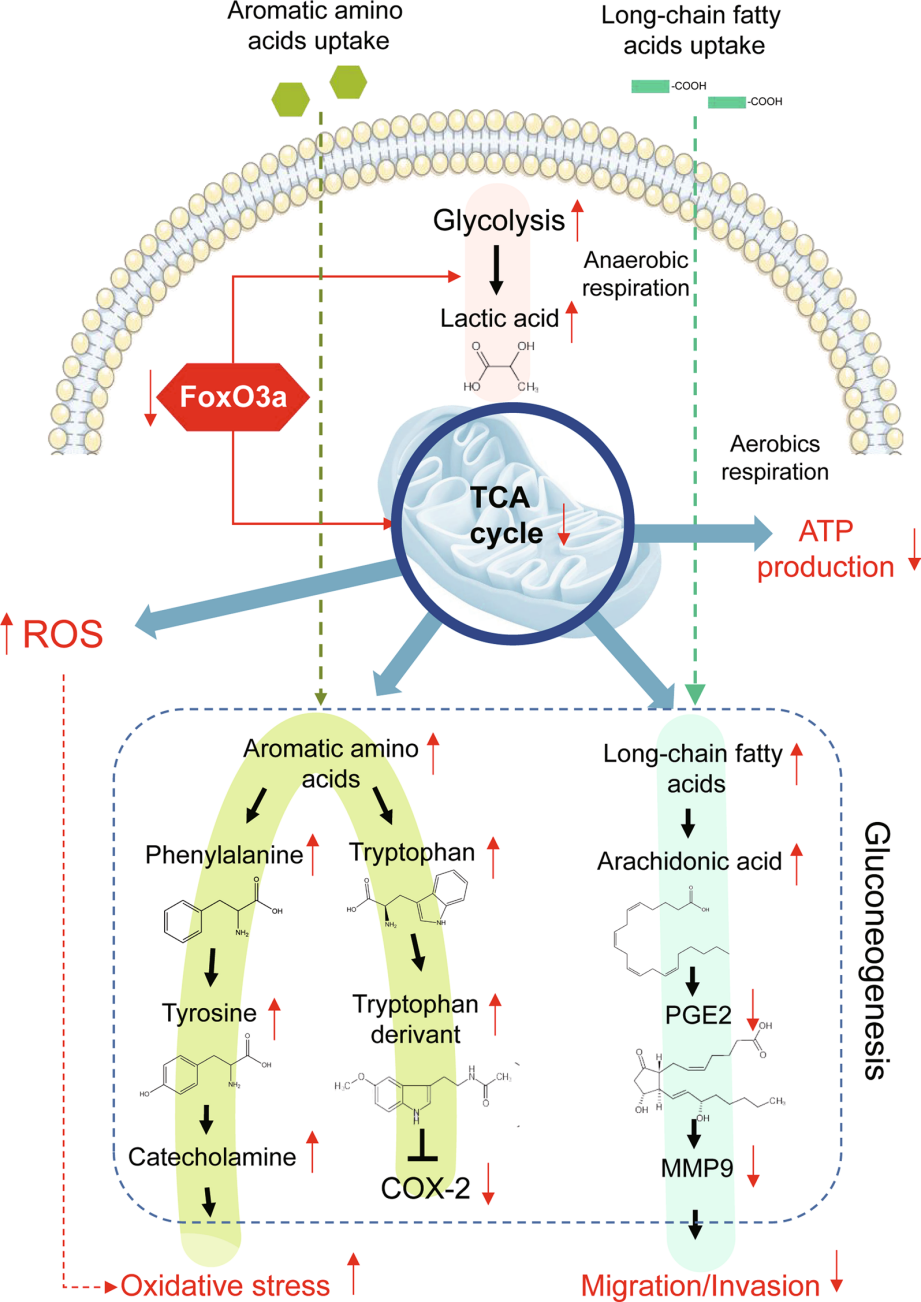
FoxO3a plays a role in metabolic remodeling and transcriptional reprogramming

In light of our study, we unmasked a novel role of FoxO3a in the metabolic remodeling and transcriptional reprogramming of early placental development, as illustrated in Fig. 7. FoxO3a exerts an essential effect on trophoblast migration and invasion owing to the regulations of COX2, MMP9, aromatic amino acids, energy metabolism, and oxidative stress. There were some limitations in this study, and future studies should explore the causative relationship between metabolic and oxidative change in cell mobility. Further investigation on primary trophoblast cells and gene knockout mice should be undertaken to support the findings in this study.

## Data Availability

All of the data generated in this study are illustrated in this article.

## Abbreviations

FoxO3a: Forkhead box O3a protein
GC–MS: Gas-chromatography-mass spectrometry
PE: Preeclampsia
FGR: Fetal growth restriction
Foxes: Forkhead protein factor family
AA: Arachidonic acid
COX-2: Cyclooxygenase-2
PGE2: Prostaglandin E2
SNP: Sodium nitroprusside
MCF: Methyl chloroformate
PLS-DA: Partial least squares discriminant analysis
ROC: Receiver operating characteristic
KEGG: Kyoto encyclopedia of genes and genomes
FDR: False discovery rate
PAPi: Pathway activity profiling
OCR: Oxygen consumption rate
PTGS2: Prostaglandin-endoperoxide synthase 2
HKDC1: Hexokinase domain containing 1
MMP9: Matrix metallopeptidase 9
5-MTP: 5-Methoxytryptophan
ALA: A-linolenic acid
